# Efficacy of acetamiprid and fipronil fly baits against the housefly (*Musca domestica* L.) under laboratory conditions

**DOI:** 10.14202/vetworld.2018.953-958

**Published:** 2018-07-16

**Authors:** Mikhail Alekseevich Levchenko, Elena Anatol’evna Silivanova, Ruzilya Khusanovna Bikinyaeva and Galina Fedorovna Balabanova

**Affiliations:** All-Russian Scientific Research Institute of Veterinary Entomology and Arachnology – Branch of Federal State Institution, Federal Research Center, Tyumen Scientific Center of Siberian Branch of the Russian Academy of Sciences, Tyumen, Russian Federation

**Keywords:** fly bait, housefly management, insecticide interaction, insecticide mixture

## Abstract

**Background:**

The housefly *Musca domestica* L. (Diptera: Muscidae) is permanent pests in livestock facilities. High fly density in livestock and poultry farms can increase the risks of economic loss and public health. Treatment with toxic baits is one of the methods for housefly control. However, development of resistance to insecticides makes it difficult to manage of flies. Anti-resistance strategies include the use of multiple pesticides with different modes of action.

**Aim:**

This study was conducted to estimate the efficacy of neonicotinoid acetamiprid and phenylpyrazole fipronil, applied alone or in the mixture, against adults of *M. domestica* and to evaluate the efficacy of fly bait formulations containing acetamiprid and fipronil under laboratory conditions.

**Materials and Methods:**

The adult flies, *M. domestic*a of laboratory strain, were used in laboratory bioassays. The efficacy of acetamiprid and fipronil as technical substances, when applied alone and in the mixture, against adult flies was tested by no-choice feeding bioassays. The insecticidal efficacy of bait formulations (wet powder) with acetamiprid or fipronil or their mixture was tested against flies by choice feeding bioassays. The probit analysis was used to calculate lethal concentrations of insecticides, and the χ^2^ test was used to analyze the interaction between fipronil and acetamiprid in the mixture.

**Results:**

Fipronil was more toxic to adults of *M. domestica* than acetamiprid in laboratory tests. Lethal concentrations for 50% mortality (95% confidence interval) of flies were 0.0159% (0.0124-0.0205) of acetamiprid and 0.000119% (0.000039-0.0002640) of fipronil. The mixture containing fipronil at concentration 0.005% and acetamiprid at concentration 0.05% had the additive effect on fly mortality.

**Conclusion:**

The results of laboratory feeding bioassays indicate that the mixture of fipronil and acetamiprid might have a potential to use in toxic bait formulations against houseflies.

## Introduction

Disinsectization of buildings is one of the obligatory procedures on livestock and poultry farms. Among other insects, non-biting flies including the housefly *Musca domestica* L. (Diptera: Muscidae) are permanent pests in livestock facilities [1,2]. The houseflies need to be controlling because their density increase is associated with the risks of economic loss and public health [3]. The housefly is known as a vector of animal and human pathogens [3-5]. In addition, the role of flies in a distribution of antibiotic resistance microorganism has been discussed [6,7]. Treatment with insecticides by sprays and toxic baits is commonly used methods for housefly control in livestock. Bait formulations have advantages, for example, easy to use, minimal risks to the environment, and other [8,9]. Over time, the insecticides used are becoming ineffective because flies develop resistance to both contact (sprayed) insecticides [10-13] and toxic baits [8,14].

Anti-resistance strategies include the use of multiple pesticides with different modes of action [15,16]. Recently, investigations have shown that insecticide mixtures may be useful for insecticide resistance management [17,18]. A combination of active ingredients (AIs) with different modes of action allows achieving a high insecticidal efficacy with simultaneous decrease of AI concentrations by the synergism effect [19]. For houseflies, Khan *et al*. [20] reported about a possibility of the use of binary (with two AIs) mixtures to control houseflies, which developed a resistance to one of the AIs. However, fly bait formulations consist of only one of just four AIs in Russian Federation: Methomyl, thiamethoxam, zeta-cypermethrin, and azamethiphos. Thus, the development of novel and effective fly baits is important.

Acetamiprid belongs to neonicotinoids that are nicotinic acetylcholine receptor agonists [21,22] and are known as relatively quick neuroactive insecticides, and the speed of action is an important characteristic of fly baits [23]. Phenylpyrazole fipronil belongs to an antagonist of γ-aminobutyric acid-gated chloride channels [21,22], has a slower insecticide effect than acetamiprid, and has not used for housefly control in Russia. According to Khan *et al*. [24], fipronil may have a potential to housefly control.

The objective of this study was to estimate the efficacy of neonicotinoid acetamiprid and phenylpyrazole fipronil, applied alone or in the mixture, against adults of *M. domestica* under laboratory conditions. The efficacy of fly bait formulations containing acetamiprid and fipronil was evaluated as well.

## Materials and Methods

### Ethical approval

Ethical approval is not applicable for such type of study.

### Study area and experimental design

Laboratory bioassays were carried out in All-Russian Scientific Research Institute of Veterinary Entomology and Arachnology - Branch of Federal State Institution, Federal Research Center, Tyumen Scientific Center of Siberian Branch of the Russian Academy of Sciences.

The experiments include laboratory tests of the insecticidal efficacy of acetamiprid and fipronil as technical substances when applied alone or in the mixture, in a no-choice feeding bioassay and the insecticidal efficacy of bait formulations (wet powder) with acetamiprid and fipronil in a choice feeding bioassay.

3-5-day-old adults (without division by sex) of *M. domestica* of a laboratory strain were used for laboratory tests. The laboratory strain of *M. domestica* was obtained from Novosibirsk Agrarian University in 2009 and was kept in the insectarium without contact with insecticides for more than 50 generations. The adult flies were reared at 26-28°C, at 50-60% relative humidity, and a 12:12 light: dark period in 25 cm×25 cm×25 cm metal cages, covered with a fine mesh. Rearing cages were supplied with glucose and milk powder (1:1 by weight), and cotton wicks lowered in cups with water.

### Preparation of fly baits

The industrial acetamiprid (97%, King Quenson Industry Group Ltd., China) and fipronil (97%, King Quenson Industry Group Ltd., China) were used as AIs. To design bait formulations, (Z)-9-tricosene (97%, Zhangzhou Enjoy Agriculture Technology Co., Ltd., China), sucrose (Sibtechnology Co., Russia), and cold swelling starch (Sibtechnology Co., Russia) were used as well. The bait formulations were wet powders and included the following components: AI - acetamiprid A (1.5%) or fipronil F (0.15%) or their mixture (A 0.15% plus F 0.015%), auxiliary agent - cold swelling starch (18%), sex attractant - tricosene (0.15%), and food attractant - sucrose (the rest of the powder). Detailed preparation of fly baits is described in the patent RU 2646044 [25]. A commercial neonicotinoid bait with thiamethoxam (“Agita 10% WG,” NOVARTIS ANIMAL HEALTH Inc., Switzerland) was used as the reference due to the lack of commercial baits with fipronil.

### No-choice feeding bioassay

The no-choice feeding bioassay was used for laboratory tests of the acetamiprid and fipronil insecticidal efficacy against adults of *M. domestica*. Flies starved for 12 h before the tests. Acetone solutions of insecticides (0.3 mL) were used to soak the sugar cube (5.5 g), and in the control test, the sugar was treated with pure acetone in the same volume. Acetamiprid was tested at concentrations from 0.0005% to 0.5%, and fipronil was tested at concentrations from 0.00001% to 0.05%. From our previous tests, it was known that 0.5% of acetamiprid and 0.05% of fipronil led to 100% mortality of flies, while acetamiprid and fipronil concentrations of 0.25% and 0.001%, respectively, caused mortality close to 90%. Hence, two stock solutions were chosen for each insecticide (0.5% and 0.25% for acetamiprid and 0.05% and 0.001% for fipronil) with a 10-fold sequential dilution for each. The mixture consisted of 0.05% of acetamiprid and 0.5% of fipronil and was serially diluted with acetone 10-fold. After the acetone evaporated, the sugar was placed in glass cups with starved flies (from 15 to 25). The cups were sealed with mesh pistons from the top and supplied with water drinkers. The mortality of the flies was recorded after 24 h. Each concentration was tested at least 3 times, and the tests were carried out on different days.

### Choice feeding bioassay

The efficacy of acetamiprid and fipronil baits was tested by a method of evaluating the effectiveness of toxic baits for fly control [26], in a choice feeding bioassay. The first bait consisted 1.5% of acetamiprid, the second bait consisted 0.15% of fipronil, and the third bait was binary and included 0.15% of acetamiprid and 0.015% of fipronil. The baits were diluted with water in a ratio of 1:3 (e.g., 10g of powder and 30g of cold water) under constant stirring. The resulting thick mass was applied in the volume of 2.5 mL on glass pieces of 100 cm^2^ by a paintbrush. The commercial “Agita 10% WG” was diluted with water according to the manufacturer’s recommendations as follows: 100 g of the product in 80 mL of water to treatment 2400 cm^2^ of a surface, and this corresponds 0.417 g of AI/100 cm^2^. Thus, a water solution of “Agita 10% WG” with concentrations of AI at 16.7% was prepared and applied to the glass piece (100 cm^2^) in the volume of 2.5 mL. The bait matrix containing starch, Z-9-tricosene, and sucrose at the same proportion was used as additional control and was applied at the same rate as bait formulations. After drying at room temperature, the treated glasses were placed in a cage of 25 cm×25 cm×25 cm; as an alternative, the conventional food was placed in the cage (glucose mixed with dry milk). Then, from 100 to 250 flies were released into the cage and were kept at temperature 25±2°C, at 50-60% relative humidity, and a 12:12 light: dark period. The insect mortality was recorded after 24 and 48 h. All laboratory tests were carried out in 3-6 replicates.

### Statistical analysis

The dose-response mortality in no-choice feeding bioassays was analyzed by probit regression analysis to calculate lethal concentrations for 50% (LC_50_) and 99% (LC_99_) mortality for 95% confidence interval. Interaction pattern (synergistic, additive, and antagonistic) of fipronil and acetamiprid tested alone and in the mixture was analyzed by the χ^2^ test [27]. The observed mortalities due to fipronil (M_F_) and acetamiprid (M_A_) were used to calculate the expected mortality M_E_ for the mixture by the formula M_E_=M_F_+M_A_(1-M_F_/100). The observed M_FA_ and expected M_E_ mortalities for the fipronil+acetamiprid mixture were used to calculate the χ^2^ value by the formula χ^2^=(M_FA_-M_E_)^2^/M_E_. If the calculated χ^2^ values exceeded the χ^2^ table value for one degree of freedom 3.841 [27], the interaction between fipronil and acetamiprid was considered to be non-additive. A positive or negative difference between M_FA_ and M_E_ indicated synergism or antagonism, respectively. The results of bait formulation tests (in a choice feeding bioassay) were analyzed by summary statistics to calculate the arithmetic mean and standard error of the mean. Statistical analysis was performed using MedCalc Software version 18.2.1.

## Results

### No-choice feeding bioassay

The mortality of flies depended on the concentrations of fipronil and acetamiprid when applied separately on sugar in a no-choice feeding bioassay (Table-1). Fipronil at the concentration of 0.005% and 0.05% and acetamiprid at the concentration of 0.5% led to the highest mortality of flies. LC_50_ of fipronil to *M. domestica* was 100-fold lower than that concentration of acetamiprid (Table-1). Such a difference between the concentrations of fipronil and acetamiprid persisted for higher mortality, for example, 99%. The slope value for acetamiprid was 1.9-fold lower than for fipronil.

Feeding of sugar with the mixture of acetamiprid and fipronil had a dose-dependent effect on the mortality of flies in a no-choice feeding bioassay (Table-2). The mortality observed for the mixture with 0.005% of fipronil and 0.05% of acetamiprid coincided with the expected value (Table-2). A non-additive effect was determined by χ^2^ analysis for the mixture diluted 10-fold. The difference between the mortality observed (M_FA_) and the mortality expected (M_E_) indicates that interaction between fipronil and acetamiprid was antagonistic for the mixture with 0.0005% of fipronil and 0.005% of acetamiprid and synergistic for the mixture with 10-fold low concentrations of insecticides (Table-2).

**Table-1:** Mortality of adult *M. domestica* (mean±SEM) in the laboratory no-choice feeding bioassay depending on acetamiprid and fipronil concentrations.

Acetamiprid			Fipronil		

Concentration (%)	Number of flies	Mortality (%)	Concentration (%)	Number of flies	Mortality (%)
0.5	80	100±0	0.05	40	100±0
0.25	43	93.15±1.87	0.005	60	100±0
0.05	96	80.90±4.13	0.001	160	89.88±3.56
0.005	81	21.28±6.52	0.0005	98	90.00±4.74
0.0025	73	11.23±5.24	0.0001	77	27.75±10.20
0.0005	70	0±0	0.00005	80	20.08±5.43
-	-	-	0.00001	75	19.93±6.28
0 (Control)	100	0±0	0 (control)		0±0
Probit results					
LC_50_ (CI 95%)	0.0159 (0.0124-0.0205)			0.000119 (0.000039-0.000264)	
LC_99_ (CI 95%)	0.451 (0.272-0.877)			0.0057 (0.0015-0.2176)	
Slope±SE	2.88±0.24			5.44±0.37	
χ^2^	358.5			318.8	
*df*	1			1	
p	<0.0001			<0.0001	

*M. domestica*=*Musca domestica*, SEM=Standard error of the mean, CI=Confidence interval, LC_50_=Lethal concentrations for 50%

**Table-2:** Mortality (mean±SEM) of adults *M. domestica* and the interaction of acetamiprid and fipronil in the laboratory no-choice feeding bioassay.

Treatment	Measurement	Mortality (%)	χ^2^	Effect
F 0.05% + A 0.5%	Observed	100	nd	nd
F 0.005% + A 0.05%	Observed	100	0	Additive
	Expected	100		
F 0.0005% + A 0.005%	Observed	68.31±1.67	6.109	Antagonistic
	Expected	92.02		
F 0.00005% + A 0.0005%	Observed	35.00±5.00	11.25	Synergistic
	Expected	20		
F 0.000005% + A 0.00005%	Observed	10.67±0.33	nd	nd
Control	Observed	0±0	nd	nd

A=Acetamiprid, F=Fipronil, nd=Not determined, Observed=Mortality due to the mixture of fipronil and acetamiprid; Expected=Mortality calculated from the observed mortality due to fipronil and acetamiprid applied separately. A χ^2^ value exceeded 3.841 (df=1, a=0.05) is considered to be synergistic or antagonistic; otherwise, it is considered to be additive, *M. domestica*=*Musca domestica*, SEM=Standard error of the mean

### Choice feeding bioassay

When acetamiprid and fipronil were tested as bait formulations, the cumulative mortality of flies was more than 90% after 24 h and achieved 100% after 48 h exposure to insecticides in choice feeding bioassay (Table-3). The bait formulation containing the mixture of fipronil and acetamiprid at 10-fold low concentrations led to 100% mortality of flies after 24 h exposure to the bait. The reference formulation (with thiamethoxam as an AI) also caused 100 mortality of flies over the same exposure time (Table-3).

**Table-3:** Insecticidal efficacy acetamiprid and fipronil baits against adults *M. domestica* in the laboratory choice feeding bioassay.

Bait formulation	Final concentration of AI	Number of flies	Mortality	

			24 h	48 h
A 1.5%	0.5%	1100	91.7 ± 0.2	100 ± 0
F 0.15%	0.05%	900	95.0 ± 0.3	100 ± 0
F 0.015% + A 0.15%	F 0.005% + A 0.05%	1200	100 ± 0	100 ± 0
Agita 10% WG	Thiamethoxam 16.7%	300	100 ± 0	100 ± 0
Bait matrix	0	600	25.8 ± 2.5	38.7 ± 3.5
Control	0	1000	0.3 ± 0.3	0.6 ± 0.4

A = Acetamiprid, F = Fipronil, AI = Active ingredient, *M. domestica = Musca domestica*

## Discussion

The present study was carried out to estimate the efficacy of acetamiprid and fipronil when applied alone and in mixture against adults of *M. domestica* in feeding bioassay and to evaluate their potential use in toxic bait formulations for housefly control. The results of no-choice feeding bioassay showed that fipronil and acetamiprid had a high insecticidal effect on adult flies that depended on insecticide concentrations. The slope value for acetamiprid was lower compared to that for fipronil that indicated a low rate of change in toxicity with an increase in the acetamiprid concentration.

Lethal doses of insecticides were calculated to compare to results obtained by other researchers because *M. domestica* strains used in our and other studies are different. The LC_50_ values by the probit analysis indicate that fipronil is 134 times more toxic to flies than acetamiprid in a feeding bioassay. Other investigations confirmed the high toxicity of fipronil to flies in laboratory no-choice feeding tests. In a Farooq and Freed research [28], the difference between LC_50_ values of fipronil and acetamiprid was 195 times. According to literature data, acetamiprid median lethal doses [29,30] converted to percentages may reach 0.00136% and 0.00032% for the susceptible strain of *M. domestica* in a laboratory feeding bioassay after 48 and 72 h exposure adult flies to insecticide, respectively. For fipronil, median lethal doses [31,32] converted to percentages vary from 0.00002% to 0.000201% for adult flies of the susceptible strain of *M.domestica* in 72 h after exposure to insecticide in feeding bioassay. The obtained and cited results show that, for some insecticides, LC_50_ values may depend on observation period after exposure. For example, LC_50_ of acetamiprid in our 24-h tests was higher than in 48- or 72-h tests by other researchers and opposite LC_50_ of fipronil in our 24-h tests was close to that in 72-h tests. Since the LD values of acetamiprid and fipronil in our tests were closed to data from other studies, our *M. domestica* strain was decided as susceptible to insecticides we tested.

According to the χ^2^ test, fipronil and acetamiprid had different interaction pattern in the mixture in a no-choice feeding bioassay. In Khan *et al*. [20] work, the mixtures of fipronil with pyrethroids had the different effect (additive, antagonistic, or synergistic) in feeding bioassay with flies of laboratory and field strains of *M. domestica*. Despite the synergistic effect determined for the mixture with low concentrations of fipronil (0.00005%) and acetamiprid (0.0005%), this mixture was not selected for further studies due to non-sufficient insecticidal effect (35% mortality). When insecticide concentrations were 10-fold higher, the antagonistic effect on fly mortality was determined. The observed mortality (100% of flies) due to the insecticide mixture containing fipronil at a concentration of 0.005% and acetamiprid at a concentration of 0.05% was the same as the expected mortality. This mixture had an additive interaction between fipronil and acetamiprid according to the χ^2^ test. It is likely that fipronil acted as the main component and masked an insecticidal activity of acetamiprid. However, the combination of fipronil and acetamiprid might be useful in a practical point of view. On the one hand, the mixture of fipronil with acetamiprid may be helpful for control of the houseflies resistant to fipronil. According to Khan *et al*. [20], resistance to fipronil was found among field populations of houseflies. On the other hand, the mixture of insecticides may be effective to delay or prevent resistance development [16]. In addition, the need for fipronil baits evaluating at livestock farms was proposed by the researchers [24].

As no-choice feeding bioassay indicated the potential of fipronil and acetamiprid for use in toxic baits against houseflies, the next stage of the study was to prepare the bait formulations consisting fipronil or acetamiprid or their mixture. Sugar is known as a good phagostimulant for houseflies, and it has been a usual component of toxic bait formulations [24]. Another standard component in commercial fly baits is the sex attractant (*Z*)-9-tricosene that increases fly catch [23]. The (*Z*)-9-tricosene concentration was chosen based on our previous experiments (unpublished). We checked the attractive effect and the weight change of sugar treated with acetone solutions of (*Z*)-9-tricosene at a concentration of 0.01% and 0.05%. The sugar cubs treated with (*Z*)-9-tricosene and pure acetone as control were placed for 24 h into rearing cages with flies mixed sex. Sugar, treated with (*Z*)-9-tricosene at a concentration of 0.05%, was more attractive and more eaten by flies than another was. For baits, the final (*Z*)-9-tricosene concentration is 0.05% in final, ready-to-use, solution preparing from the bait formulation. To achieve this (*Z*)-9-tricosene concentration, the content of this attractant in the formulations (in powders) was increased 3-fold, i.e., up to 0.15%. Some commercial baits are containing more tricosene (e.g., Interflytox Köder 0.6%).

Cold swelling starch was chosen as an auxiliary agent that made the final solution of baits thick and easy to use in practice. Starch likely has an insecticidal effect, and in our control experiment with the matrix, the death of flies was observed. A possible reason for this is that the starch adheres to insect covers (according to own experience) and probably falls into the spiracle, preventing normal breathing of insects. Thus, our fly toxic bait formulations include insecticide, sucrose, (*Z*)-9-tricosene, and cold swelling starch and were made as wet powders [25]. For insecticides, their concentrations in bait formulations (wet powders) were increased 3-fold because the way of use involves the dilution of powder with water; as a result, the insecticide concentration will decrease in final (ready-to-use) solution.

The laboratory choice feeding bioassay showed a sufficient efficacy of prepared bait formulations against adults *M. domestica* (>90% mortality). For comparison, according to Li *et al*. [33], the cumulative mortality of houseflies was 68.76% after 24 h exposure to the commercial fly bait containing 0.5% of imidacloprid (another neonicotinoid) in choice feeding tests. Murillo *et al*. [9] reported 62.1% and 87.8% fly mortality for the susceptible strain of *M. domestica* after 1 and 2 days of exposure to the commercial bait containing 0.5% of imidacloprid in choice feeding tests, respectively. Interesting, that fly mortality for the field strain of *M. domestic*a was only 4.3% and 6.8% due to this commercial bait for the same conditions [9]. Importantly, the insecticidal efficacy of the bait with the mixture of fipronil and acetamiprid was not less than the efficacy of baits containing only fipronil or acetamiprid although the binary bait included 10-fold low concentrations of these insecticides. A decrease in pesticide concentrations while maintaining high efficacy is an important task of the pesticide sciences because it can reduce the pesticide load on the environment [34].

## Conclusion

The present results demonstrated that acetamiprid and fipronil, separately or in the mixture, as a part of fly toxic baits had a high insecticidal efficacy against adult houseflies under laboratory conditions. The benefit of the mixture of fipronil and acetamiprid is a reduced concentration of AIs. We suggest that fipronil and acetamiprid, separately or in combination, have a potential to use in toxic bait formulations and such formulations might be appropriate to incorporate into rotation schemes of insecticides for housefly management in livestock. However, there is a need for the future laboratory tests with field populations of *M. domestica* and field trials on assessing the efficacy of bait formulations in livestock or poultry farms.

## Author’s Contributions

MAL and EAS designed the study and analyzed the data. RKB and GFB reared the laboratory strain of *M. domestica* and carried out the laboratory tests. MAL prepared fly bait formulations and tested them under laboratory conditions. EAS participated in the laboratory tests and wrote the paper. All authors read and approved the final manuscript.
